# Supplementary material for: Immune-Mediated Renal Diseases: A Team-Based Learning Module for Preclinical Medical Students
Source: MedEdPORTAL. 2021 Dec 16;17:11206. doi: 10.15766/mep_2374-8265.11206 (PMC8674152; doi:10.15766/mep_2374-8265.11206)
Supplement: Supplementary file 1 — Student Instructions.docxiRAT & tRAT - Student Version.docxiRAT & tRAT - Instructor Version.docxTeam Application Activities - Student Version.docxTeam Application Activities - Instructor Version.docxPostsession Survey.docx [file mep_2374-8265.11206-s001.zip › A. Student Instructions.docx]

**Learning Objectives:**

1. Describe the immunopathogenic mechanisms of renal disease.
2. Categorize immunologic injury to the renal system.
3. Describe the clinical presentation and diagnosis of acute nephritic syndromes.

**Required Reading:**

Harrison’s Principles of Internal Medicine, 20e

- Chapter 308: Glomerular Diseases

Sections: Introduction, Pathogenesis of Glomerular Disease, Progression of Glomerular Disease, Renal Pathology, Acute Nephritic Syndromes

- Chapter 311: Vascular Injury to the Kidney

Sections: Thrombotic Microangiopathy, Hemolytic-Uremic Syndrome
