# Supplementary material for: Immune-Mediated Renal Diseases: A Team-Based Learning Module for Preclinical Medical Students
Source: MedEdPORTAL. 2021 Dec 16;17:11206. doi: 10.15766/mep_2374-8265.11206 (PMC8674152; doi:10.15766/mep_2374-8265.11206)
Supplement: Supplementary file 1 — Student Instructions.docxiRAT & tRAT - Student Version.docxiRAT & tRAT - Instructor Version.docxTeam Application Activities - Student Version.docxTeam Application Activities - Instructor Version.docxPostsession Survey.docx [file mep_2374-8265.11206-s001.zip › B. iRAT & tRAT - Student Version.docx]

**Immune-Mediated Renal Diseases Team-Based Learning Module – iRAT and tRAT**

ATTENTION, STUDENTS: If you are accessing this material BEFORE it is used in your course, please do NOT read this document prior to the class session. An answer key is included in this module, which is designed to lead you through a learning experience that reinforces your knowledge of the content. Early review or dissemination of this material to others will diminish the learning opportunity and be considered academic misconduct.

1. A 25-year-old male presents to your office with hematuria 3 days after the onset of a productive cough and fever. Urinalysis reveals 20 – 40 erythrocytes per high powered field, red blood cell casts, and 2 + proteinuria. Following renal biopsy, immunofluorescence shows granular mesangial IgA deposits associated with mesangial hypercellularity. Which of the following is the most likely diagnosis in this patient?
2. Granulomatosis with polyangiitis (Wegener’s)
3. IgA nephropathy (Berger Disease)
4. Membranoproliferative glomerulonephritis
5. Goodpasture’s syndrome
6. Systemic lupus erythematosus
7. A 23-year-old male presents with complaints of hemoptysis and dysuria. BP is 160/100 mm Hg, serum blood urea nitrogen and creatinine are elevated, and urinalysis shows hematuria and RBC casts. A 24-hour urine excretion yields 1 gm/day protein. A kidney biopsy is obtained, and immunofluorescence shows linear IgG staining in the glomeruli. Which of the following antibodies is likely pathogenic for this patient’s disease?
8. Anti-glomerular basement membrane antibody
9. Anti-neutrophil perinuclear antibody
10. Anti-neutrophil cytoplasmic antibody
11. Anti-dsDNA antibody
12. Anti-phospholipid antibody
13. A 5-year-old girl is admitted to the floor with a several day history of bloody diarrhea.  Other members of the daycare she attends also had bloody diarrhea. She is irritable and lethargic. Her skin has also turned slightly yellow. Her arms have multiple petechiae. Lab results show creatinine of 4.0 mg/dL (ref 0.6-1.2 mg/dL), platelet of 40,000/mm^3^ (ref 150,000-400,000/ mm^3^), and hemoglobin of 7 g/dL (ref 10.5-13.5 g/dL). What is the mechanism of action of the toxin that mediates the pathogenesis of this disease?
    1. The A subunit activates adenylate cyclase
    2. The A subunit inactivates the 60S ribosome
    3. The A subunit inactivates G proteins
    4. The B subunit inhibits neurotransmitter release
    5. It acts as a superantigen
14. A 3-year-old boy presents with a 7-day history of jaundice, abdominal pain, and watery diarrhea that became bloody after the first day. He has become lethargic and has not been eating or drinking. Three days before the onset of symptoms, he had visited the county fair with his family and had eaten a hamburger. His vital signs are as follows: T 38.5 C, HR 135, BP 82/54. Physical examination is significant for petechiae on his legs and diffuse abdominal tenderness to palpation. Lab-work shows BUN 72 mg/dL (7-18 mg/dL) and creatinine 8.1 mg/dL (ref 0.6-1.2 mg/dL). A peripheral blood shows schistocytes. Which of the following would likely be an additional lab finding?
15. Decreased liver enzymes
16. High ANA titer
17. Elevated serum IgA
18. Low platelet count
19. Atypical lymphocytes on smear
20. Most autoimmune mechanisms that lead to damage of glomeruli are:
21. NK cell mediated responses
22. T_h_1-directed cell mediated responses
23. Cytotoxic T lymphocyte-directed responses
24. TLR directed responses
25. Humoral immune responses
26. Three weeks after recovering from pharyngitis, an 82-year-old male with lung cancer presents to his primary physician with periorbital edema and dark brown urine. Renal biopsy demonstrates glomerular infiltrates of neutrophils, subendothelial immune deposits, and subepithelial deposits. Which of the following is likely found in this patient?
    1. High anti-dsDNA levels in serum
    2. Atypical lymphocytes on blood smear
    3. High IgA serum titers
    4. Mutations in collagen type IV
    5. High serum anti-streptococcal antibody levels
27. Which of the following immune mechanisms underlies the pathogenesis of type I membranoproliferative glomerulonephritis (MPGN)?
    1. Primary Immunodeficiency
    2. Secondary Immunodeficiency
    3. Type II Hypersensitivity
    4. Type III Hypersensitivity
    5. Allergy
