# Supplementary material for: Immune-Mediated Renal Diseases: A Team-Based Learning Module for Preclinical Medical Students
Source: MedEdPORTAL. 2021 Dec 16;17:11206. doi: 10.15766/mep_2374-8265.11206 (PMC8674152; doi:10.15766/mep_2374-8265.11206)
Supplement: Supplementary file 1 — Student Instructions.docxiRAT & tRAT - Student Version.docxiRAT & tRAT - Instructor Version.docxTeam Application Activities - Student Version.docxTeam Application Activities - Instructor Version.docxPostsession Survey.docx [file mep_2374-8265.11206-s001.zip › C. iRAT & tRAT - Instructor Version.docx]

**Immune-Mediated Renal Diseases Team-Based Learning Module – iRAT and tRAT**

ATTENTION, STUDENTS: If you are accessing this material BEFORE it is used in your course, please do NOT read this document prior to the class session. An answer key is included in this module, which is designed to lead you through a learning experience that reinforces your knowledge of the content. Early review or dissemination of this material to others will diminish the learning opportunity and be considered academic misconduct.

1. A 25-year-old male presents to your office with hematuria 3 days after the onset of a productive cough and fever. Urinalysis reveals 20 – 40 erythrocytes per high powered field, red blood cell casts, and 2 + proteinuria. Following renal biopsy, immunofluorescence shows granular mesangial IgA deposits associated with mesangial hypercellularity. Which of the following is the most likely diagnosis in this patient?
2. Granulomatosis with polyangiitis (Wegener’s)
3. IgA nephropathy (Berger Disease)
4. Membranoproliferative glomerulonephritis
5. Goodpasture’s syndrome
6. Systemic lupus erythematosus

*Answer = b: IgA Nephropathy (Berger Disease) is characterized by the presence of prominent IgA deposits in the mesangial region, mesangial proliferation, and recurrent hematuria. A recent infection is known to trigger symptom onset. Options a, c, and e would not present with the presence of IgA deposits. Goodpasture’s syndrome can be ruled out because it typically presents with hemoptysis and is associated with linear IgG staining (rarely IgA) as a result of antibodies to the glomerular basement membrane.*

1. A 23-year-old male presents with complaints of hemoptysis and dysuria. BP is 160/100 mm Hg, serum blood urea nitrogen and creatinine are elevated, and urinalysis shows hematuria and RBC casts. A 24-hour urine excretion yields 1 gm/day protein. A kidney biopsy is obtained, and immunofluorescence shows linear IgG staining in the glomeruli. Which of the following antibodies is likely pathogenic for this patient’s disease?
2. Anti-glomerular basement membrane antibody
3. Anti-neutrophil perinuclear antibody
4. Anti-neutrophil cytoplasmic antibody
5. Anti-dsDNA antibody
6. Anti-phospholipid antibody

*Answer = a: The patient described in the question stem is presenting with Goodpasture’s syndrome which is characterized by acute renal failure and hemoptysis. In Goodpasture’s syndrome, linear IgG staining is the classic pattern seen when viewing immunofluorescence of glomeruli in affected patients as a result of autoantibodies directed against glomerular basement membrane antigens. The other options would not be associated with a linear staining pattern on immunofluorescence. Options d and e are associated with the diffuse proliferative glomerulonephritis of systemic lupus erythematosus. Microscopic polyangiitis is associated with anti-neutrophil perinuclear antibody (option b) and granulomatosis with polyangiitis is associated with anti-neutrophil cytoplasmic antibody (option c).*

1. A 5-year-old girl is admitted to the floor with a several day history of bloody diarrhea.  Other members of the daycare she attends also had bloody diarrhea. She is irritable and lethargic. Her skin has also turned slightly yellow. Her arms have multiple petechiae. Lab results show creatinine of 4.0 mg/dL (ref 0.6-1.2 mg/dL), platelet of 40,000/mm^3^ (ref 150,000-400,000/ mm^3^), and hemoglobin of 7 g/dL (ref 10.5-13.5 g/dL). What is the mechanism of action of the toxin that mediates the pathogenesis of this disease?
   1. The A subunit activates adenylate cyclase
   2. The A subunit inactivates the 60S ribosome
   3. The A subunit inactivates G proteins
   4. The B subunit inhibits neurotransmitter release
   5. It acts as a superantigen

*Answer = b: The patient described in the question stem is suffering from hemolytic-uremic syndrome which is characterized by platelet-fibrin thrombi deposition in the glomerular capillaries. Children and young adults are at highest risk and present with bleeding manifestations and thrombocytopenia. The thrombi produce microvascular occlusions that cause tissue ischemia and organ dysfunction. Most cases occur following intestinal infection with strains of Escherichia coli that produce Shiga-like toxins. The A subunit of Shiga toxin cleaves an adenine from the host cell’s 28S rRNA, irreversibly inhibiting ribosomal function and leading to endothelial cell apoptosis. The A subunit of cholera toxin activates adenylate cyclase (option a). Toxins produced by Clostridium species inactivate G proteins. The B subunit of botulinum toxin binds to its cell surface receptor and the A subunit consists of the enzyme that inhibits neurotransmitter release (option d). Superantigens are associated with staphylococcal and streptococcal infections (option e).*

1. A 3-year-old boy presents with a 7-day history of jaundice, abdominal pain, and watery diarrhea that became bloody after the first day. He has become lethargic and has not been eating or drinking. Three days before the onset of symptoms, he had visited the county fair with his family and had eaten a hamburger. His vital signs are as follows: T 38.5 C, HR 135, BP 82/54. Physical examination is significant for petechiae on his legs and diffuse abdominal tenderness to palpation. Lab-work shows BUN 72 mg/dL (7-18 mg/dL) and creatinine 8.1 mg/dL (ref 0.6-1.2 mg/dL). A peripheral blood shows schistocytes. Which of the following would likely be an additional lab finding?
2. Decreased liver enzymes
3. High ANA titer
4. Elevated serum IgA
5. Low platelet count
6. Atypical lymphocytes on smear

*Answer = b: The young patient described in the question stem is suffering from hemolytic-uremic syndrome which is characterized by platelet-fibrin thrombi deposition in the glomerular capillaries. Widespread consumption of platelets leads to a low platelet count. The thrombi create flow abnormalities that shear red blood cells, producing a microangiopathic hemolytic anemia. Most cases follow intestinal infection caused by ingestion of ground meat contaminated with strains of Escherichia coli that produce Shiga-like toxins. Options a and c through e include laboratory findings that would not coincide with an infection by Shiga-toxin producing Escherichia coli.*

1. Most autoimmune mechanisms that lead to damage of glomeruli are:
2. NK cell mediated responses
3. T_h_1-directed cell mediated responses
4. Cytotoxic T lymphocyte-directed responses
5. TLR directed responses
6. Humoral immune responses

*Answer = e: Humoral immune responses underlie most forms of primary glomerulopathy and many of the secondary glomerular disorders. Glomerular deposits of immunoglobulins, often with components of complements, are found in the majority of individuals with glomerulonephritis. Cell-mediated immune responses usually occur as a result of antibody-instigated injury (option a). NK cell mediated responses and cytotoxic T lymphocyte-directed responses are defenses against intracellular pathogens and cancer (options b and c). TLR directed responses are part of the innate immune system and important in detecting pathogens (option d).*

1. Three weeks after recovering from pharyngitis, an 82-year-old male with lung cancer presents to his primary physician with periorbital edema and dark brown urine. Renal biopsy demonstrates glomerular infiltrates of neutrophils, subendothelial immune deposits, and subepithelial deposits. Which of the following is likely found in this patient?
   1. High anti-dsDNA levels in serum
   2. Atypical lymphocytes on blood smear
   3. High IgA serum titers
   4. Mutations in collagen type IV
   5. High serum anti-streptococcal antibody levels

*Answer = e:* *The patient described in the question stem is suffering from poststreptococcal glomerulonephritis which is associated with the influx of leukocytes caused by the in situ formation of immune complexes containing streptococcal antigens and anti-streptococcal antibodies. The latent period between infection and onset of nephritis is compatible with the time required for the production of antibodies and the formation of immune complexes. Elevation of anti-streptococcal antibody titers is an important laboratory finding. Options a through d include findings that would not coincide with postinfectious glomerulonephritis.*

1. Which of the following immune mechanisms underlies the pathogenesis of type I membranoproliferative glomerulonephritis (MPGN)?
   1. Primary Immunodeficiency
   2. Secondary Immunodeficiency
   3. Type II Hypersensitivity
   4. Type III Hypersensitivity
   5. Allergy

*Answer = d: Type I membranoproliferative glomerulonephritis is characterized by deposition of immune complexes containing IgG and complement which describes a type III hypersensitivity reaction. Type II hypersensitivity reactions are caused by an antibody that binds to a fixed cell or tissue surface antigen. Options a, b and e do not underlie the pathogenesis of any of the glomerulonephritides.*
