# Supplementary material for: Immune-Mediated Renal Diseases: A Team-Based Learning Module for Preclinical Medical Students
Source: MedEdPORTAL. 2021 Dec 16;17:11206. doi: 10.15766/mep_2374-8265.11206 (PMC8674152; doi:10.15766/mep_2374-8265.11206)
Supplement: Supplementary file 1 — Student Instructions.docxiRAT & tRAT - Student Version.docxiRAT & tRAT - Instructor Version.docxTeam Application Activities - Student Version.docxTeam Application Activities - Instructor Version.docxPostsession Survey.docx [file mep_2374-8265.11206-s001.zip › D. Team Application Activities - Student Version.docx]

**Immune-Mediated Renal Diseases Team-Based Learning Module – Application Activity #1**

ATTENTION, STUDENTS: If you are accessing this material BEFORE it is used in your course, please do NOT read this document prior to the class session. An answer key is included in this module, which is designed to lead you through a learning experience that reinforces your knowledge of the content. Early review or dissemination of this material to others will diminish the learning opportunity and be considered academic misconduct.

1. A 10-year-old male presents to the ED with petechiae on his legs. His initial lab results are:

| **Test** | **Value** | **Reference** |
| --- | --- | --- |
| Hemoglobin | 10.5 g/dL | 10.5-13.5 g/dL |
| Platelets | 45,000/μL | 150,000-450,000/μL |
| BUN | 43 mg/dL | 7-18 mg/dL |
| Creatinine | 2.46 mg/dL | 0.6-1.2 mg/dL |
| Lactate dehydrogenase | 5455 U/L | 45-90 U/L |

Possible etiologies at this point are (choose all that apply):

1. Henoch-Schonlein purpura
2. Hemolytic-uremic syndrome
3. Atypical hemolytic-uremic syndrome
4. Thrombotic thrombocytopenic purpura (TTP)
5. Pneumococcal pneumonia
6. IgA nephropathy

Correct answers include: B, C, D, and E

2. Which of the following would be expected on a peripheral blood smear?

1. Rouleaux formation
2. Schistocytes
3. Spur cells
4. Giant cells
5. Atypical lymphocytes

Correct answer is B

3. What is the appropriate empirical antimicrobial while awaiting culture results?

1. Ciprofloxacin PO
2. Trimethoprim-sulfamethoxazole PO
3. Ceftriaxone IV
4. Azithromycin PO
5. No antibiotics should be given

Correct answer is E

4. Which of the following cell types does Shiga toxin target (choose all that apply)?

1. Red blood cells
2. Parietal epithelial cells
3. Colonic epithelial cells
4. Glomerular endothelial cells
5. Mesangial cells
6. Monocytes
7. Podocytes
8. Platelets

Correct answers are: B, C, D, F, and H

5. Gallery Walk: Diagram the immune mechanisms that lead to cell injury as a result of Shiga toxin.^1^

**Immune-Mediated Renal Diseases Team-Based Learning Module – Application Activity #2**

| **Disease**  **and**  **Type of Hypersensitivity** | **Most Affected Population?** | **Underlying Immune Mechanisms** | **Diagnostic Features** |
| --- | --- | --- | --- |
| **Primary membranous glomerulonephritis**^2^**,**^3^ |  |  |  |
| **Anti-glomerular basement membrane (GBM) disease**^2^**,**^3^ |  |  |  |
| **Lupus glomerulonephritis**^2^**,**^3^ |  |  |  |
| **Post-streptococcal**  **Glomerulonephritis**^4^ |  |  |  |
| **IgA nephropathy**^2^ |  |  |  |
| **Type I membranoproliferative glomerulonephritis**^2^**,**^5^**,**^6^ |  |  |  |
| **Henoch-Schonlein purpura (IgA vasculitis)**^7,8^ |  |  |  |
| **ANCA small-vessel vasculitis**^3^**,**^9^ |  |  |  |
| **Tubulointerstitial nephritis**^10^**,**^11^**,**^12^ |  |  |  |

**References**

1. Moake JL. Thrombotic microangiopathies. *N Engl J Med.* 2002;347(8):589-600.

2. Tecklenborg J, Clayton D, Siebert S, Coley SM. The role of the immune system in kidney disease. *Clin Exp Immunol.* 2018;192(2):142-150.

3. Mastroianni-Kirsztajn G, Hornig N, Schlumberger W. Autoantibodies in renal diseases - clinical significance and recent developments in serological detection. *Front Immunol.* 2015;6:221.

4. Nasr SH, Fidler ME, Valeri AM, et al. Postinfectious glomerulonephritis in the elderly. *J Am Soc Nephrol.* 2011;22(1):187-195.

5. Kupin WL. Viral-Associated GN: Hepatitis C and HIV. *Clin J Am Soc Nephrol.* 2017;12(8):1337-1342.

6. Fervenza F, Sethi, S. Evaluation and treatement of membranoproliferative glomerulonephritis. In: *UpToDate.*2019.

7. Kauffmann RH, Herrmann WA, Meyer CJ, Daha MR, Van Es LA. Circulating IgA-immune complexes in Henoch-Schonlein purpura. A longitudinal study of their relationship to disease activity and vascular deposition of IgA. *Am J Med.* 1980;69(6):859-866.

8. Kiryluk K, Moldoveanu Z, Sanders JT, et al. Aberrant glycosylation of IgA1 is inherited in both pediatric IgA nephropathy and Henoch-Schonlein purpura nephritis. *Kidney Int.* 2011;80(1):79-87.

9. Kallenberg CG. Pathophysiology of ANCA-associated small vessel vasculitis. *Curr Rheumatol Rep.* 2010;12(6):399-405.

10. Shawar R, Patamasucon P, Rowles S. Case 2: Fever and Back Pain in 13-year-old Girl. *Pediatr Rev.* 2017;38(1):46-47.

11. Praga M, Appel, GB. Clinical manifestations and diagnossis of acute interstitial nephritis. In: Palevsky P, ed. *UpToDate.* UpToDate, Waltham, MA2018:30.

12. Praga M, Gonzalez E. Acute interstitial nephritis. *Kidney Int.* 2010;77(11):956-961.
