# Supplementary material for: Immune-Mediated Renal Diseases: A Team-Based Learning Module for Preclinical Medical Students
Source: MedEdPORTAL. 2021 Dec 16;17:11206. doi: 10.15766/mep_2374-8265.11206 (PMC8674152; doi:10.15766/mep_2374-8265.11206)
Supplement: Supplementary file 1 — Student Instructions.docxiRAT & tRAT - Student Version.docxiRAT & tRAT - Instructor Version.docxTeam Application Activities - Student Version.docxTeam Application Activities - Instructor Version.docxPostsession Survey.docx [file mep_2374-8265.11206-s001.zip › E. Team Application Activities - Instructor Version.docx]

**Immune-Mediated Renal Diseases Team-Based Learning Module – Application Activity #1**

ATTENTION, STUDENTS: If you are accessing this material BEFORE it is used in your course, please do NOT read this document prior to the class session. An answer key is included in this module, which is designed to lead you through a learning experience that reinforces your knowledge of the content. Early review or dissemination of this material to others will diminish the learning opportunity and be considered academic misconduct.

1. A 10-year-old male presents to the ED with petechiae on his legs. His initial lab results are:

| **Test** | **Value** | **Reference** |
| --- | --- | --- |
| Hemoglobin | 10.5 g/dL | 10.5-13.5 g/dL |
| Platelets | 45,000/μL | 150,000-450,000/μL |
| Blood urea nitrogen | 43 mg/dL | 7-18 mg/dL |
| Creatinine | 2.46 mg/dL | 0.6-1.2 mg/dL |
| Lactate dehydrogenase | 5455 U/L | 45-90 U/L |

Possible etiologies at this point are (choose all that apply):

1. Henoch-Schonlein purpura
2. Hemolytic-uremic syndrome
3. Atypical hemolytic-uremic syndrome
4. Thrombotic thrombocytopenic purpura (TTP)
5. Pneumococcal pneumonia
6. IgA nephropathy

*The correct answers include B, C, D, and E. All conditions listed in B through E can induce thrombotic microangiopathy which is characterized by petechiae on presentation, a low platelet count, and high blood urea nitrogen, creatinine, and lactate dehydrogenase levels. Pneumococcal-associated hemolytic uremic syndrome (HUS) is the second most common infectious cause of HUS, second to strains of Escherichia coli that produce Shiga toxins. It occurs mainly in young children and infants who usually present with pneumonia. IgA nephropathy can result in high creatinine, but the other labs would be normal as patients with IgA nephropathy do not exhibit bleeding manifestations such as petechiae and thrombocytopenia. The clinical triad associated with Henoch-Schonlein purpura is palpable purpura (without thrombocytopenia), abdominal pain, and arthritis.*

2. Which of the following would be expected on a peripheral blood smear?

1. Rouleaux formation
2. Schistocytes
3. Spur cells
4. Giant cells
5. Atypical lymphocytes

*The correct answer is B. Thrombotic microangiopathy (TMA) is characterized by endothelial cell injury in the terminal arterioles and capillaries. Platelet and hyaline thrombi causing partial or complete occlusion are integral to the histopathology of TMA. In this patient, TMA is accompanied by microangiopathic hemolytic anemia as evidenced by thrombocytopenia and the presence of schistocytes, fragmented red blood cells, on peripheral blood smear.*

3. What is the appropriate empirical antimicrobial while awaiting culture results?

1. Ciprofloxacin PO
2. Trimethoprim-sulfamethoxazole PO
3. Ceftriaxone IV
4. Azithromycin PO
5. No antibiotics should be given

*The correct answer is E. Antibiotics are not recommended for hemolytic uremic syndrome due to Shiga toxin-producing Escherichia coli or pneumococcal pneumonia because there is inadequate proof that they are beneficial, and in fact a number of studies have shown an association with increased risk of hemolytic-uremic syndrome.*

4. Which of the following cell types does Shiga toxin target (choose all that apply)?

1. Red blood cells
2. Parietal epithelial cells
3. Colonic epithelial cells
4. Glomerular endothelial cells
5. Mesangial cells
6. Monocytes
7. Podocytes
8. Platelets

*The correct answers are B, C, D, E, F, and H. Shiga toxin-producing Escherichia coli cause hemorrhagic colitis and hemolytic-uremic syndrome (HUS). Following enterohemorrhagic E. coli colonization of the colon, Shiga toxin enters the circulation and travels on the surface of platelets or monocytes to the kidneys where it attaches to molecules on glomerular capillary endothelial cells, mesangial cells, and parietal epithelial cells. The main reason why gastrointestinal infection particularly affects kidneys is thought to be the tissue tropism of Shiga-toxin based on the strong expression of glycolipid surface receptor globotriaosylceramide (Gb3) on cells of the renal glomeruli.*

5. Gallery Walk: Diagram the immune mechanisms that lead to cell injury as a result of Shiga toxin.^1^


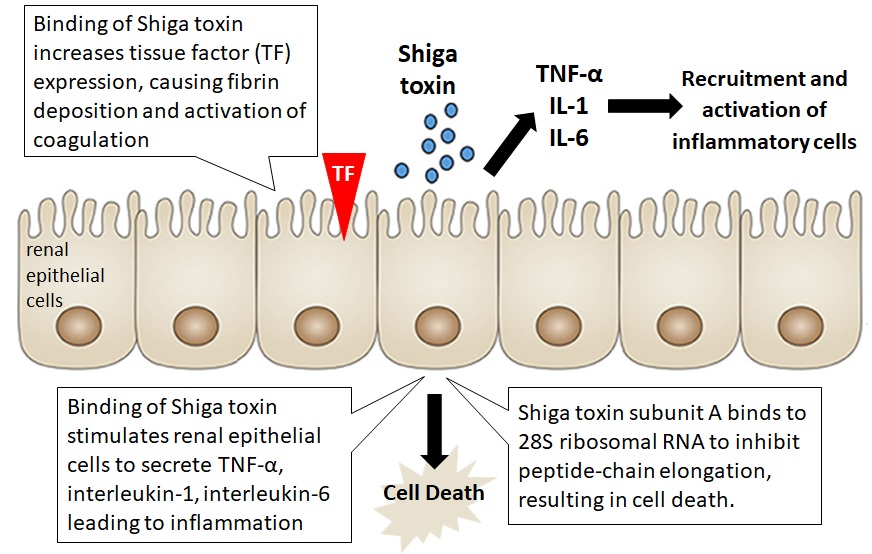


Created by authors.

**Immune-Mediated Renal Diseases Team-Based Learning Module – Application Activity #2**

| **Disease**  **and**  **Type of Hypersensitivity** | **Most Affected Population?** | **Underlying Immune Mechanisms** | **Diagnostic Features** |
| --- | --- | --- | --- |
| **Primary membranous glomerulonephritis**^2^**,**^3^  *Hypersensitivity Type: II* | - 30-50 y/o - 2:1 male to female ratio | - Autoreactive Abs to podocyte membrane proteins (most common: PLA2 receptor)   - Ab isotype: IgG - Abs activate complement, which leads to podocyte injury and remodeling of the glomerular basement membrane | - Renal biopsy:   - Thickening of basement membrane along peripheral capillary loops   - Deposition of IgG and C3 |
| **Anti-glomerular basement membrane (GBM) disease**^2^**,**^3^  **Type: Goodpasture’s syndrome**  *Hypersensitivity Type: II* | - Men 20-30 y/o - Men and women 60-70 | - Autoreactive Abs to Collagen IV α3 chain located in basement membrane   - Ab isotype: IgG | - Renal biopsy:   - Linear immunofluorescent staining of basement membrane IgG and/or complement components - Concomitant with lung hemorrhage |
| **Lupus glomerulonephritis**^2^**,**^3^  *Hypersensitivity Type: III* | - Most severe in African American female adolescents | - anti-dsDNA Abs   - Ab isotype: IgG - Immune complex deposition, complement activation, neutrophil infiltration, inflammatory reaction | - Proteinuria - anti-dsDNA antibodies |
| **Post-streptococcal**  **Glomerulonephritis**^4^  *Hypersensitivity Type: III* | - Underdeveloped   - 2-14 y/o - Developed:   - Elderly - Post impetigo:   - 2-6 weeks - Post pharyngitis: 1-3 weeks | - Abs bind Streptococcal antigens - Immune complexes form   - Ab isotype: IgM & IgG   - Antigen: streptococcal pyrogenic exotoxin B (SPEB) - Complement activation, neutrophils infiltrate, inflammatory reaction | - Hematuria, proteinuria, pyuria, red blood cell casts, edema, hypertension - Headache, malaise, anorexia, flank pain   - 50% of patients - 1st week of symptoms: decreased CH50 and C3 |

| **Disease** | **Who?** | **What?** | **Diagnostic Features** |
| --- | --- | --- | --- |
| **IgA nephropathy**^2^  *Hypersensitivity Type: III* | - Men 20-30 y/o - Onset is thought to require 2 hits  1. Mis-expressed IgA 2. Viral or other antigen exposure | - Synthesis of poorly glycosylated IgA   - Exposure of cryptic epitopes on IgA leads to anti-IgA Abs   - Ab isotype: IgG - Immune complex deposition in mesangium   - Complement activation | - Recurrent episodes of macroscopic hematuria during or right after an upper respiratory infection - Proteinuria - Renal biopsy:   - IgA positive staining - Increased serum IgA titers |
| **Type I membranoproliferative glomerulonephritis**^2^**,**^5^**,**^6^  *Hypersensitivity Type: III* | - Primary MPGN - children and young adults - Secondary to infections   - Most common: Hepatitis C   - 30% of patients acquire after ~10 yrs of viremia | - anti-HCV IgM-specific IgG   - IgG produced that binds IgM (that is specific for HCV)     - Immune complex formation, complement activation | - Proteinuria, hematuria - Decreased serum C3 and C4 levels |
| **Henoch-Schonlein purpura (IgA vasculitis)**^7,8^  *Hypersensitivity Type: III* | - Children 3-15 y/o   - Distinct from IgA neuropathy:     - Systemic symptoms, younger age, not usually preceded by infection | - Mechanism unknown - Hypothesis: anti-endothelial cell IgA leads to neutrophil migration and activation via FcαR   - Inflammation | - Hematuria - Renal biopsy   - IgA deposition in mesangium |

| **Disease** | **Who?** | **What?** | **Diagnostic Features** |
| --- | --- | --- | --- |
| **ANCA small-vessel vasculitis**^3^**,**^9^  **Type: Granulomatosis with polyangiitis (Wegener’s)**  *Hypersensitivity Type: IV* | - Age of onset ~40 - More common in patients with exposure to silica dust or alpha-1-antitrypsin deficiency | - ANCA antibodies [*a*nti*n*eutrophil *c*ytoplasmic *a*ntibodies], directed against components situated within neutrophil cytoplasmic granules, bind to and activate neutrophils in the vasculature causing them to degranulate and damage vascular walls. The most common ANCA antigen is proteinase 3 (PR3). - Pathogenesis appears to involve the IL-17/TH_17_ axis. | - Serologic demonstration of antineutrophil cytoplasmic antibodies - Chest x-ray reveals nodules & infiltrates - Biopsy shows small vessel vasculitis with adjacent noncaseating granulomas |
| **Tubulointerstitial nephritis**^10^**,**^11^**,**^12^  **Type: Drug-induced**  **[⅔ of cases]**  *Hypersensitivity Type: IV* | - Average onset: after 10 days of use - Antibiotics:   - Ampicillin, cephalosporin, ciprofloxacin, methicillin, penicillin, vancomycin - NSAIDs - Associated with inflammatory bowel disease | - Medication haptenates proteins   - Tubulointerstitial proteins: dendritic cells present haptenated peptides on MHC Class II. CD4+ T cell- mediated responses (cytokines recruit leukocytes) and damage renal cells   - Circulating proteins/molecules: β-lactam degradation product binds lysine residues on albumin. These complexes circulate and are filtered by the kidney. Dendritic cells specific for this complex activate T cells, leading to cytokine release, immune cell infiltration, inflammation, and renal damage. | - Renal biopsy:   - Inflammatory infiltrates     - T cells     - Macrophages   - Destructive fibrosis   - Hematuria, proteinuria, leukocyturia |

Bolded information was provided at the beginning of Activity 2. Unbolded information was inserted as groups revealed their findings during the 20 min report-out discussion.

**References**

1. Moake JL. Thrombotic microangiopathies. *N Engl J Med.* 2002;347(8):589-600.

2. Tecklenborg J, Clayton D, Siebert S, Coley SM. The role of the immune system in kidney disease. *Clin Exp Immunol.* 2018;192(2):142-150.

3. Mastroianni-Kirsztajn G, Hornig N, Schlumberger W. Autoantibodies in renal diseases - clinical significance and recent developments in serological detection. *Front Immunol.* 2015;6:221.

4. Nasr SH, Fidler ME, Valeri AM, et al. Postinfectious glomerulonephritis in the elderly. *J Am Soc Nephrol.* 2011;22(1):187-195.

5. Kupin WL. Viral-Associated GN: Hepatitis C and HIV. *Clin J Am Soc Nephrol.* 2017;12(8):1337-1342.

6. Fervenza F, Sethi, S. Evaluation and treatement of membranoproliferative glomerulonephritis. In: *UpToDate.*2019.

7. Kauffmann RH, Herrmann WA, Meyer CJ, Daha MR, Van Es LA. Circulating IgA-immune complexes in Henoch-Schonlein purpura. A longitudinal study of their relationship to disease activity and vascular deposition of IgA. *Am J Med.* 1980;69(6):859-866.

8. Kiryluk K, Moldoveanu Z, Sanders JT, et al. Aberrant glycosylation of IgA1 is inherited in both pediatric IgA nephropathy and Henoch-Schonlein purpura nephritis. *Kidney Int.* 2011;80(1):79-87.

9. Kallenberg CG. Pathophysiology of ANCA-associated small vessel vasculitis. *Curr Rheumatol Rep.* 2010;12(6):399-405.

10. Shawar R, Patamasucon P, Rowles S. Case 2: Fever and Back Pain in 13-year-old Girl. *Pediatr Rev.* 2017;38(1):46-47.

11. Praga M, Appel, GB. Clinical manifestations and diagnossis of acute interstitial nephritis. In: Palevsky P, ed. *UpToDate.* UpToDate, Waltham, MA2018:30.

12. Praga M, Gonzalez E. Acute interstitial nephritis. *Kidney Int.* 2010;77(11):956-961.
