# Supplementary material for: Immune-Mediated Renal Diseases: A Team-Based Learning Module for Preclinical Medical Students
Source: MedEdPORTAL. 2021 Dec 16;17:11206. doi: 10.15766/mep_2374-8265.11206 (PMC8674152; doi:10.15766/mep_2374-8265.11206)
Supplement: Supplementary file 1 — Student Instructions.docxiRAT & tRAT - Student Version.docxiRAT & tRAT - Instructor Version.docxTeam Application Activities - Student Version.docxTeam Application Activities - Instructor Version.docxPostsession Survey.docx [file mep_2374-8265.11206-s001.zip › Educational_Summary_Report_11206.pdf]

MedEdPORTAL® | The AAMC Journal of Teaching and Learning Resources

Original Publication

Open Access

Immune-Mediated Renal Diseases: A Team-Based Learning Module for Preclinical Medical Students

Michelle Demory Beckler, PhD*, Amanda J. Chase, PhD

*Corresponding author: mbeckler@nova.edu

Abstract

Introduction: The underlying immune mechanisms of renal diseases are complex, and medical students benefit from learning these complexities in the setting of team-based learning (TBL), where they can apply foundational immunologic concepts to clinical problems. **Methods:** This immune-mediated renal disease TBL module focusing on glomerular disease was delivered over a period of 2 consecutive years in an integrated cardiovascular, pulmonary, and renal block within the second semester of the first year of medical school. Each class consisted of 53 students, for a total of 106 students. The TBL module consisted of a seven-question individual and team readiness assurance test (iRAT and tRAT) and two clinically relevant team application activities. Following the TBL module, students completed an 18-item survey that collected information on student perspectives of engagement. **Results:** For the 2-year period, the iRAT average scores were 73% and 74%, and the tRAT average scores were 100% and 99%, respectively. The survey assessing student perceptions of engagement yielded positive feedback. **Discussion:** In summary, this TBL module effectively integrates the foundational and clinical sciences to teach higher-order concepts in immunology and renal disease pathogenesis to medical students.

Keywords
Team-Based Learning, Immunology, Glomerular Disease, Renal Disease, Hypersensitivity, Pathology, Nephrology

Educational Objectives

By the end of this module, learners will be able to:

- Describe the immunopathogenic mechanisms of renal disease.
- Categorize immunologic injury to the renal system.
- Describe the clinical presentation and diagnosis of acute nephritic syndromes.

Introduction

Team-based learning (TBL) is a teaching and learning modality that emphasizes individual testing and group collaboration, rather than traditional didactic teaching. Over the years, TBL has become an increasingly popular strategy for engaging undergraduate medical learners. Advantages of the TBL format are that learners can simultaneously apply their current knowledge, utilize real-time research methods to understand underlying disease mechanisms, and learn collaboratively. Additionally, learners find TBL useful because they receive immediate instructor feedback.[1,2]

Citation:
Demory Beckler M, Chase AJ. Immune-mediated renal diseases: a team-based learning module for preclinical medical students. *MedEdPORTAL.* 2021;17:11206.
https://doi.org/10.15766/mep_2374-8265.11206

Medical textbooks cover the clinical presentation, diagnosis, and treatment of renal diseases; however, the immune mechanisms that lead to glomerular injury are not always discussed in detail. Therefore, a greater emphasis is needed in a discussion-based classroom setting such as TBL where students can work through complex mechanisms and verbalize difficult concepts. This TBL module was designed for students to apply their fundamental knowledge of immune mechanisms and hypersensitivity to renal disease. Following a review of the literature, we did not find any other TBL activities that include content on nephritic syndromes and mechanisms of immune-mediated damage in glomerular diseases.

The TBL module was developed as part of the cardiovascular, pulmonary, and renal (CPR) block. In this preclinical block, medical students explore how the CPR systems regulate the body's homeostatic functions and apply this knowledge to the diagnosis and management of acute and chronic diseases. The renal portion of the block includes the role of the renal system in regulation of fluid and blood pressure, electrolyte and acid-base

Copyright © 2021 Beckler and Chase. This is an open-access publication distributed under the terms of the Creative Commons Attribution license.

balance, and renal, glomerular, vascular, and interstitial diseases. Both the style and educational objectives associated with this TBL work in synergy with the overall desired outcomes for the CPR block.

Methods

Curricular Context

This TBL module was developed as part of the CPR block, a 12-week block in the second semester of the first year at an allopathic medical school. By the beginning of this block, students have acquired foundational knowledge in basic sciences and clinical application skills. For immunology in particular, students have a working understanding of the innate and adaptive immune responses as well as hypersensitivities and the application of immunology to pathology, pharmacology, microbiology, and physiology. CPR is the last block of the first year, and similar to the other organ-system blocks, it is taught in a multidisciplinary fashion. Content is delivered through a variety of instructional approaches, including didactic sessions, TBL, small-group case-based learning, human structure and function laboratories, and simulation experiences. The curriculum is heavily grounded in an inquiry-based, sequential reasoning approach to complex disease processes. Large-group didactic and case-based learning sessions throughout the block reinforce concepts in basic immunology, hypersensitivity, and pathology of renal diseases. In addition to summative individual and team readiness assurance tests (iRAT and tRAT), formative quizzes are assigned weekly, and there are NBME-style summative assessments that occur periodically throughout the block. To ensure parallel delivery of information with minimal duplication and seamless integration across all disciplines, we consulted teaching faculty, and their curricular content was referenced in the design of this TBL. We delivered this TBL module in both 2019 and 2020 to first-year medical students.

Team Formation

At the beginning of their first semester, our medical college places the 53 first-year medical students into 10 TBL teams, consisting of five to six members each, and these teams stay together for the entirety of the first semester. At the beginning of their second semester, the students are placed into new teams that remain together for the entirety of the second semester. The preclerkship director determines the composition of the team members to maximize gender balance and team diversity. We conducted this TBL module during the second semester. Human subjects research approval was obtained from the Nova Southeastern University Institutional Review Board.

Advance Preparation Resources

We provided the learning objectives and reading assignments to the students at least 1 week prior to the TBL module. Students were expected to prepare by reading sections of Chapter 308,[3] Glomerular Diseases, and Chapter 311,[4] Vascular Injury to the Kidney, from the 20th edition of *Harrison's Principles of Internal Medicine* (Appendix A). All students had access to the required textbook through the university's health professions library website.

Readiness Assurance Process and Immediate Feedback

For the 2019 and 2020 modules, the same readiness assurance processes were used. We gave the iRAT at the beginning of the TBL module (Appendices B and C). The iRAT consisted of first- and second-order questions that were written to assess the students' understanding of the prereading assignment. We gave students 10 minutes to complete the seven-question, closed-resource test, which we administered on Canvas. We did not immediately provide students with their scores after the iRAT. Following completion of the iRAT, the tRAT was administered (Appendices B and C). Teams worked together to answer the same multiple-choice questions as in the iRAT. The tRAT used Immediate Feedback Assessment Technique cards to provide immediate feedback about the correct answer to each question. We awarded teams full credit for each question if the correct answer was selected on the first attempt; otherwise, points were deducted progressively with each additional attempt to find the correct answer. We gave teams 15 minutes to complete the seven-question, closed-resource tRAT. Following the tRAT, we facilitated a discussion of student questions that had not been resolved during the tRAT team discussions.

Team Application Activities

The application phase of the TBL contained two multipart team application activities (Appendices D and E). The first activity entailed sequential delivery of five clinical scenario questions that explored the differential diagnosis, pathogenesis, and management of a patient presenting with signs and symptoms of renal injury. The first four questions were multiple choice, and the last question prompted students to produce a diagram display during a gallery walk. Our gallery walk required students to view, at stations either in person or via Zoom (see Online Delivery via Zoom and InteDashboard section, below), other teams' diagrams and to vote on their favorite. The second activity consisted of an open-ended problem set in which teams utilized a table to describe the classification, epidemiology, pathogenesis, and diagnosis of nine immune-mediated renal diseases.

Copyright © 2021 Beckler and Chase. This is an open-access publication distributed under the terms of the Creative Commons Attribution license.

We used two modalities to deliver this TBL module. In the first year, we used live, in-person delivery supplemented with Canvas. In the spring of 2020, we delivered the module online as part of a university-wide shift to online education during the COVID-19 pandemic. Therefore, in the second year, we implemented online delivery via Zoom, utilizing the breakout room feature supplemented with InteDashboard.

Facilitation Schema

- Readiness assurance tests (Appendices B and C):
  - iRAT (10 minutes).
  - tRAT (15 minutes).
  - Clarification and discussion (15 minutes).
- Team application activities (Appendices D and E):
  - Activity 1: teams and discussion (20 minutes).
  - Activity 1: gallery walk (5 minutes).
  - Activity 2: teams (15 minutes).
  - Activity 2: discussion (20 minutes).

During activity 1 in the team application activities, we presented students with a clinical scenario on hemolytic uremic syndrome to encourage understanding of differential diagnosis and underlying causes of immune-mediated diseases. Because each question in activity 1 built upon an appropriate interpretation of the previous question, questions 1-5 of this activity were delivered sequentially (Appendices D and E). For example, students discussed question 1 in their teams followed by a large-group simultaneous report-out before moving to question 2. For question 1, we provided the students with a brief patient presentation and laboratory values for a patient presenting with petechiae, low platelets, and increased markers of impaired kidney function. Subsequent questions delved further into the disease process and the mechanisms of Shiga toxin–induced, immune-mediated cell injury. Activity 1 culminated in each team producing a diagram of mechanisms leading to Shiga toxin–induced cell injury. All of the diagrams were displayed, and a gallery walk ensued. See Appendix E for an example diagram.

During activity 2 in the team application activities, we emphasized the immunopathogenesis of renal diseases. Here, we asked teams to cooperatively research, describe, and discuss nine immune-related renal diseases. We provided the class with a table with the disease name in the left-most column (Appendix D) and asked teams to classify the disease-associated type of hypersensitivity, to determine which population would be most affected by the disease, to analyze the underlying immune mechanisms, and to identify diagnostic features. Teams took 15 minutes to research the diseases. Then, teams were chosen randomly during the 20-minute simultaneous report-out discussion to reveal their findings. During this time, we electronically shared a master table with the class, and we acted as scribes to fill in the blank cells of the table as teams reported and discussed their findings. By the end of activity 2, the class had developed a completed table that could be used as a guide to study the nine immune-related renal diseases. See Appendix E for an instructor version of the completed table.

Of the nine immune-related renal diseases, students appeared to struggle with the underlying immune mechanisms of two. These were type I membranoproliferative glomerulonephritis and IgA nephropathy. For the former, students often described type II membranoproliferative glomerulonephritis rather than type I. For the latter, students typically required additional prompting to identify the specific antigen and the antibody isotype involved in the type III hypersensitivity reaction. Large-group discussion of these two diseases provided an excellent opportunity to analyze complex underlying immunopathogenic disease processes in a clinically relevant context.

Online Delivery via Zoom and InteDashboard

In our spring 2020 delivery of the TBL module, we used online delivery following established best practices for online TBL.[5] First, students took the iRAT individually via Canvas with Respondus LockDown Browser. Then, students joined a Zoom session where they were sent into breakout rooms to take the tRAT via InteDashboard with their TBL teams. We used InteDashboard to monitor team progression. Once the teams had finished the tRAT, all teams were brought back to the main room for clarification. A similar process was followed for the team application activities in which teams were sent to breakout rooms to discuss each individual activity and enter their answers into InteDashboard. We, as facilitators, jumped from room to room to listen to the team discussions. Subsequently, the teams were brought back to the main Zoom room for large-group discussion. InteDashboard includes a gallery walk feature that was utilized for activity 1.

Evaluation Strategy

After the TBL module, we administered an 18-question, postsession survey that gauged students' perceptions of their confidence in the TBL content, individual and team contributions, and overall enjoyment (Appendix F). The questions were adapted from the Assessing Student Perspective of Engagement in Class Tool.[6] Student responses were recorded using a 5-point Likert scale. A free-response item was also provided that allowed students to offer feedback on the module.

Copyright © 2021 Beckler and Chase. This is an open-access publication distributed under the terms of the Creative Commons Attribution license.

Results

Over a period of 2 consecutive years, a total of 106 students, forming 20 teams, participated in this TBL module. The class averages for the TBL were similar each year, even with a switch to online delivery in 2020. For the 2019 in-person module, the averages were 73% on the iRAT and 100% on the tRAT. For the 2020 online module, the averages were 74% on the iRAT and 99% on the tRAT. The iRAT and tRAT questions were first- and second-order and were answerable from the preassigned reading.

Student engagement during the module was compelling, with students reporting an increased understanding of the immune mechanisms contributing to renal disease. In addition, a majority of students agreed or strongly agreed with statements that the TBL content "integrates more than one discipline" and "helps me fill gaps in knowledge" (Appendix F and Figure). Approximately 75% of students participated in the postsession survey. At the conclusion of the CPR block, students completed five NBME-style examination questions based on this TBL module. The average score on these summative questions was 91% in 2019 and 87% in 2020.

Discussion

Immunology and its application to human health and disease states can be challenging for medical students. In our experience, applying basic and advanced immunology concepts to clinical scenarios yields maximum synthesis and integration of information. Here, we have described a TBL module that employs a disease-based approach to encourage student research and drive critical analysis of immune-mediated disease processes. Outcomes of this module were collected over the 2 consecutive years of its delivery and show not only student success on assessments but also a high level of student satisfaction.

We believe a crucial aspect of the module's success was the backward design used in its creation and refinement.[7]

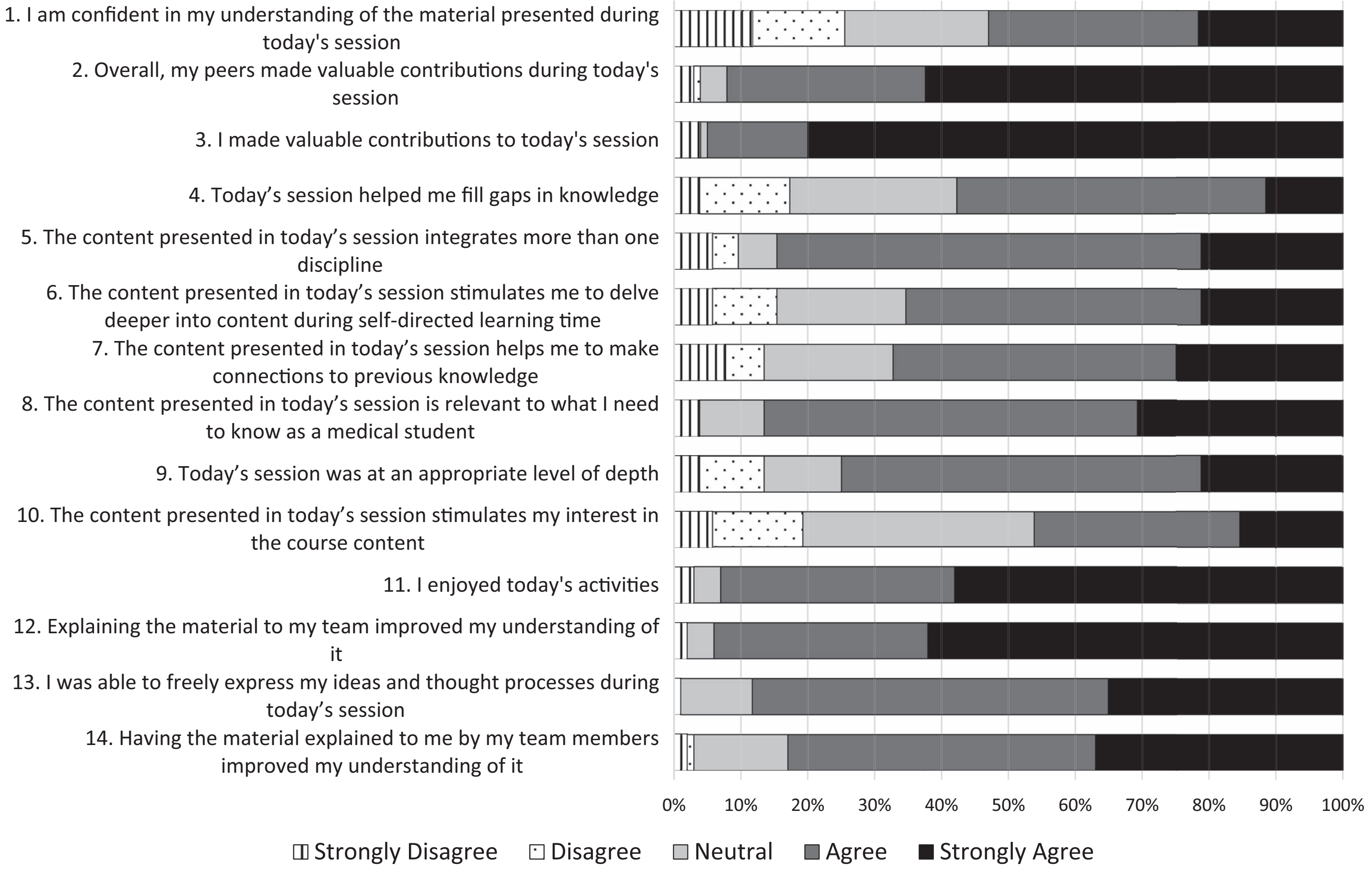

Figure. Student responses to Likert-scale questions on the postsession survey.

Copyright © 2021 Beckler and Chase. This is an open-access publication distributed under the terms of the Creative Commons Attribution license.

Beyond the learning objectives, our major aims for the TBL module were (1) to enhance critical analysis of evidence-based resources to help guide application of pathologic immunology and hypersensitivity reactions (activities 1 and 2) and (2) to develop an application experience in which the students would produce a table of immunological conditions and their underlying mechanisms that they could use to prepare for future assessments (activity 2). With these goals, the two application activities were created first, followed by the readiness assurance test, which was designed to assess comprehension of the reading and to supplement the application activities. The application activities were intentionally designed so that students would be required to go beyond reliance on textbook reading and USMLE review books to research, synthesize, and analyze primary literature to answer parts of the activity.

While the TBL module was deemed a success in its first year of delivery, an important lesson was learned. We modified activity 2 for the second year to include fewer disease states. This allowed ample time for each team to simultaneously analyze all nine disease states and contribute to the large-group discussion. Consequently, groups fact-checked each other, as well as debating and discussing underlying disease mechanisms, with little to no clarification needed from us, as facilitators.

With the university-wide shift to online education in the second year of delivery, we mindfully modified the TBL module to maximize the effectiveness of team distance learning. Of particular importance was activity 2, the research-based application activity, because the use of breakout rooms for team discussion meant that the two of us, as faculty facilitators, would not be immediately available while the teams navigated through the activity. The purposeful use of Zoom breakout rooms and InteDashboard report-out features helped us, as facilitators, to monitor team responses to the team application activities in real time. In addition, the mindful use of these online modalities helped foster student engagement and intimate team dynamics. Indeed, outcomes from the postmodule survey and block assessment demonstrated that the online TBL delivery was a success.

In today's academic climate, it is vital to develop flexible activities that can be administered in both in-person and online settings. This flexible, multimode-delivery TBL module resulted in a high level of student satisfaction with the session itself and its incorporation of multiple disciplines as well as success on the block assessment exam. We noted high student engagement at both the in-person 2019 and online 2020 modules, leading us to conclude that students not only found the session enjoyable and useful but also performed well when assessed on the material. However, one limitation of this study is that more data are needed for both in-person and online delivery to determine whether there are true outcome differences based on the delivery method. In the future, we anticipate augmenting the module with additional gallery walks based on diagrams of the higher-order immune mechanisms involved in renal disease.

Appendices

A. Student Instructions.docx
B. iRAT & tRAT - Student Version.docx
C. iRAT & tRAT - Instructor Version.docx
D. Team Application Activities - Student Version.docx
E. Team Application Activities - Instructor Version.docx
F. Postsession Survey.docx

All appendices are peer reviewed as integral parts of the Original Publication.

Michelle Demory Beckler, PhD: Assistant Professor, Department of Medical Education, Nova Southeastern University Dr. Kiran C. Patel College of Allopathic Medicine; ORCID: https://orcid.org/0000-0002-0244-0005

Amanda J. Chase, PhD: Associate Professor, Department of Medical Education, Nova Southeastern University Dr. Kiran C. Patel College of Allopathic Medicine; ORCID: https://orcid.org/0000-0003-0761-3769

Disclosures
None to report.

Funding/Support
None to report.

Ethical Approval
The Nova Southeastern University Institutional Review Board deemed further review of this project not necessary.

References

1. Parmelee D, Michaelsen LK, Cook S, Hudes PD. Team-based learning: a practical guide: AMEE Guide no. 65. *Med Teach*. 2012;34(5):e275-e287. https://doi.org/10.3109/0142159X.2012.651179
2. Michaelsen LK, Sweet M. The essential elements of team-based learning. *New Dir Teach Learn*. 2008;2008(116):7-27. https://doi.org/10.1002/tl.330

Copyright © 2021 Beckler and Chase. This is an open-access publication distributed under the terms of the Creative Commons Attribution license.

3. Lewis JB, Neilson EG. Glomerular diseases. In: Jameson JL, Fauci AS, Kasper DL, Hauser SL, Longo DL, Loscalzo J, eds. *Harrison's Principles of Internal Medicine*. 20th ed. McGraw-Hill Education; 2018:chap 308.

4. Go RS, Leung N. Vascular injury to the kidney. In: Jameson JL, Fauci AS, Kasper DL, Hauser SL, Longo DL, Loscalzo J, eds. *Harrison's Principles of Internal Medicine*. 20th ed. McGraw-Hill Education; 2018:chap 311.

5. Clark M, Merrick L, Styron J, et al. Off to on: best practices for online team-based learning. Team-Based Learning Collaborative. January 15, 2018. Updated August 16, 2018. http://www.teambasedlearning.org/wp-content/uploads/2018/08/Off-to-On_OnlineTBL_WhitePaper_ClarkEtal2018_V3.pdf

6. Wiggins BL, Eddy SL, Wener-Fligner L, et al. ASPECT: a survey to assess student perspective of engagement in an active-learning classroom. *CBE Life Sci Educ.* 2017;16(2):ar32. https://doi.org/10.1187/cbe.16-08-0244

7. Wiggins G, McTighe J. *Understanding by Design*. Association for Supervision and Curriculum Development; 1998.

Received: April 6, 2021
Accepted: September 15, 2021
Published: December 16, 2021

Copyright © 2021 Beckler and Chase. This is an open-access publication distributed under the terms of the Creative Commons Attribution license.
