# Supplementary material for: Immune-Mediated Renal Diseases: A Team-Based Learning Module for Preclinical Medical Students
Source: MedEdPORTAL. 2021 Dec 16;17:11206. doi: 10.15766/mep_2374-8265.11206 (PMC8674152; doi:10.15766/mep_2374-8265.11206)
Supplement: Supplementary file 1 — Student Instructions.docxiRAT & tRAT - Student Version.docxiRAT & tRAT - Instructor Version.docxTeam Application Activities - Student Version.docxTeam Application Activities - Instructor Version.docxPostsession Survey.docx [file mep_2374-8265.11206-s001.zip › F. Postsession Survey.docx]

**Immune-Mediated Renal Diseases Team-Based Learning Module – Post-Session Survey**

1. I am confident in my understanding of the material presented during today's session.

🞎 Strongly Disagree 🞎 Disagree 🞎 Neutral 🞎 Agree 🞎 Strongly Agree

1. Overall, my peers made valuable contributions during today's session.

🞎 Strongly Disagree 🞎 Disagree 🞎 Neutral 🞎 Agree 🞎 Strongly Agree

1. I made valuable contributions to today's session.

🞎 Strongly Disagree 🞎 Disagree 🞎 Neutral 🞎 Agree 🞎 Strongly Agree

1. Today’s session helped me fill gaps in knowledge.

🞎 Strongly Disagree 🞎 Disagree 🞎 Neutral 🞎 Agree 🞎 Strongly Agree

1. The content presented in today’s session integrates more than one discipline.

🞎 Strongly Disagree 🞎 Disagree 🞎 Neutral 🞎 Agree 🞎 Strongly Agree

1. The content presented in today’s session stimulates me to delve deeper into content during self-directed learning time.

🞎 Strongly Disagree 🞎 Disagree 🞎 Neutral 🞎 Agree 🞎 Strongly Agree

1. The content presented in today’s session helps me to make connections to previous knowledge.

🞎 Strongly Disagree 🞎 Disagree 🞎 Neutral 🞎 Agree 🞎 Strongly Agree

1. The content presented in today’s session is relevant to what I need to know as a medical student.

🞎 Strongly Disagree 🞎 Disagree 🞎 Neutral 🞎 Agree 🞎 Strongly Agree

1. Today’s session was at an appropriate level of depth.

🞎 Strongly Disagree 🞎 Disagree 🞎 Neutral 🞎 Agree 🞎 Strongly Agree

1. The content presented in today’s session stimulates my interest in the course content.

🞎 Strongly Disagree 🞎 Disagree 🞎 Neutral 🞎 Agree 🞎 Strongly Agree

1. I enjoyed today's activities.

🞎 Strongly Disagree 🞎 Disagree 🞎 Neutral 🞎 Agree 🞎 Strongly Agree

1. Explaining the material to my team improved my understanding of it.

🞎 Strongly Disagree 🞎 Disagree 🞎 Neutral 🞎 Agree 🞎 Strongly Agree

1. I was able to freely express my ideas and thought processes during today’s session.

🞎 Strongly Disagree 🞎 Disagree 🞎 Neutral 🞎 Agree 🞎 Strongly Agree

1. Having the material explained to me by my team members improved my understanding of it.

🞎 Strongly Disagree 🞎 Disagree 🞎 Neutral 🞎 Agree 🞎 Strongly Agree

1. Following today's TBL session, I am________________in my understanding of foundational concepts.

🞎 Much more confident 🞎 More confident 🞎 Just as confident 🞎 Less confident 🞎 Much less confident

1. Following today's TBL session, I am________________ in my ability to work and contribute as a team member.

🞎 Much more confident 🞎 More confident 🞎 Just as confident 🞎 Less confident 🞎 Much less confident

1. In your previous education, were you asked to work in teams during large group sessions?

🞎 Yes 🞎 No

1. Feel free to include any comments you would like to share regarding today's TBL session.
